# Integration of acoustic and electric hearing is better in the same ear than across ears

**DOI:** 10.1038/s41598-017-12298-3

**Published:** 2017-10-02

**Authors:** Qian-Jie Fu, John J. Galvin, Xiaosong Wang

**Affiliations:** Department of Head and Neck Surgery, David Geffen School of Medicine, University of California Los Angeles, Los Angeles, California 90095 USA

## Abstract

Advances in cochlear implant (CI) technology allow for acoustic and electric hearing to be combined within the same ear (electric-acoustic stimulation, or EAS) and/or across ears (bimodal listening). Integration efficiency (IE; the ratio between observed and predicted performance for acoustic-electric hearing) can be used to estimate how well acoustic and electric hearing are combined. The goal of this study was to evaluate factors that affect IE in EAS and bimodal listening. Vowel recognition was measured in normal-hearing subjects listening to simulations of unimodal, EAS, and bimodal listening. The input/output frequency range for acoustic hearing was 0.1–0.6 kHz. For CI simulations, the output frequency range was 1.2–8.0 kHz to simulate a shallow insertion depth and the input frequency range was varied to provide increasing amounts of speech information and tonotopic mismatch. Performance was best when acoustic and electric hearing was combined in the same ear. IE was significantly better for EAS than for bimodal listening; IE was sensitive to tonotopic mismatch for EAS, but not for bimodal listening. These simulation results suggest acoustic and electric hearing may be more effectively and efficiently combined within rather than across ears, and that tonotopic mismatch should be minimized to maximize the benefit of acoustic-electric hearing, especially for EAS.

## Introduction

The coarse spectral resolution provided by cochlear implants (CIs) greatly limits performance in challenging listening conditions such as perception of speech in noise, speech prosody, vocal emotion, tonal language, music, etc.^1–8^. Adding detailed low-frequency information via acoustic hearing (aided or unaided) to electric hearing can benefit CI users’ speech and music perception^9–21^. However, some CI users do not experience significant benefits with acoustic-electric hearing^22–24^, and others experience interference between acoustic and electric hearing^20,25,26^. This suggests differences among CI users’ abilities to combine acoustic and electric stimulation patterns^27,28^.

It is unclear whether acoustic and electric stimulation patterns are best combined within the same ear or across ears. With electric-acoustic stimulation (EAS), electric hearing via CI is combined with acoustic hearing in the ipsilateral ear; several CI manufacturers combine a hearing aid with the CI processor. With bimodal hearing, electric hearing via CI is combined with acoustic hearing in the contralateral ear. For EAS patients, the current spread associated with electric stimulation may interfere with acoustic hearing at the periphery. For bimodal listeners, there is no peripheral interaction between acoustic and electric hearing, which may be advantageous. For bimodal patients, clinical fitting of the CI is often performed without regard to the extent of residual acoustic hearing. For EAS patients, the low input frequency to the CI is often adjacent to the extent of acoustic hearing. Especially for bimodal listeners, the acoustic-to-electric frequency allocation typically maximizes the amount of acoustic information within the CI. Depending on the insertion depth and extent of the electrode array, the input acoustic frequency may be lower than the characteristic frequency associated with the electrode position, resulting in tonotopic mismatch. Large tonotopic mismatches have been shown to negatively affect speech performance with the CI only^29–31^ and in CI simulations^29,32–34^. Passive learning and/or explicit training can often offset some of the deficits associated with tonotopic mismatch^35–43^. However, adaptation may not be complete, and gradual adaptation to a tonotopic mismatch may be less difficult for CI users^44,45^. Interestingly, work by Reiss and colleagues has shown that acoustic-electric pitch matches may change as bimodal CI users gain experience with their device, suggesting perceptual adaptation driven by the CI frequency allocation^46,47^. Other research has shown that acoustic-electric pitch matching can be difficult and highly variable^48,49^.

The effect of tonotopic mismatch in electric hearing is less understood in the context of acoustic-electric hearing. Some studies have compared different CI frequency allocations for bimodal and EAS patients. Reiss *et al*.^50^ found no significant difference among 3 EAS patients between a wide input frequency range (which maximized the speech information within the CI, but with some degree of tonotopic mismatch) and a clinical range in which the low CI input frequency was adjacent to the extent of acoustic hearing. Fowler *et al*.^51^ found better speech performance in bimodal listeners with good residual acoustic hearing when the low CI input frequency was increased, presumably reducing tonotopic mismatch. Peters *et al*.^49^ found no significant correlation in CI patients with single-sided deafness between the degree of tonotopic mismatch (as inferred from imaging and acoustic-electric pitch-matching) and speech performance. Gifford *et al*.^52^ reported that a broad frequency allocation did not always produce the best performance in bimodal or bimodal-EAS listening, possibly due to low-frequency tonotopic mismatch. Thus, data with real bimodal and EAS presents a somewhat confusing picture. There is likely a tradeoff between preserving speech information within the CI and reducing tonotopic mismatch between acoustic and electric hearing.

While stimulation at the correct tonotopic place is necessary for complex pitch perception^53^, other frequency components important for speech, such as vowel first formant (F1, associated with tongue height) and second formant (F2, associated with tongue position within the vocal cavity) may also be sensitive to tonotopic mismatch. This may be especially true when one component is delivered to the correct place (e.g., F1 with acoustic hearing) and another is delivered to a shifted place (e.g., F1 and/or F2 with electric hearing), resulting in interference between F1 cues and/or distortion to the ratio between F1 and F2 frequencies. It is unclear whether such distortions to speech features produce more interference with peripheral (EAS) or central processing (bimodal).

In this study, vowel recognition was measured in normal-hearing (NH) subjects listening to simulations of residual acoustic hearing and electric hearing. NH listeners and simulations were used to explicitly control the extent of stimulation within the cochlea and to directly compare perception of combined acoustic and electric hearing within and across ears. Such comparisons cannot be easily made in real EAS and bimodal CI listeners, as the extent/quality of residual acoustic hearing and the electrode-neural interface (the number and position of intra-cochlear electrodes relative to healthy neurons) is likely to vary across ears and/or patients. Vowel recognition was used to measure speech performance to explore the effects of preservation of speech information, spectral resolution (acoustic versus electric hearing) and tonotopic mismatch; sensitivity to speech features such as F1 and F2 might be reduced for sentence recognition where context cues are available. We hypothesized that vowel recognition would be similar between the EAS and bimodal simulations, as previous studies have shown that listeners are able to integrate independent information across ears^18,27,54–57^. We also hypothesized that there would be a tradeoff between the amount of speech information in the CI simulation and the degree of tonotopic mismatch.

## Results

**Figure 1:**
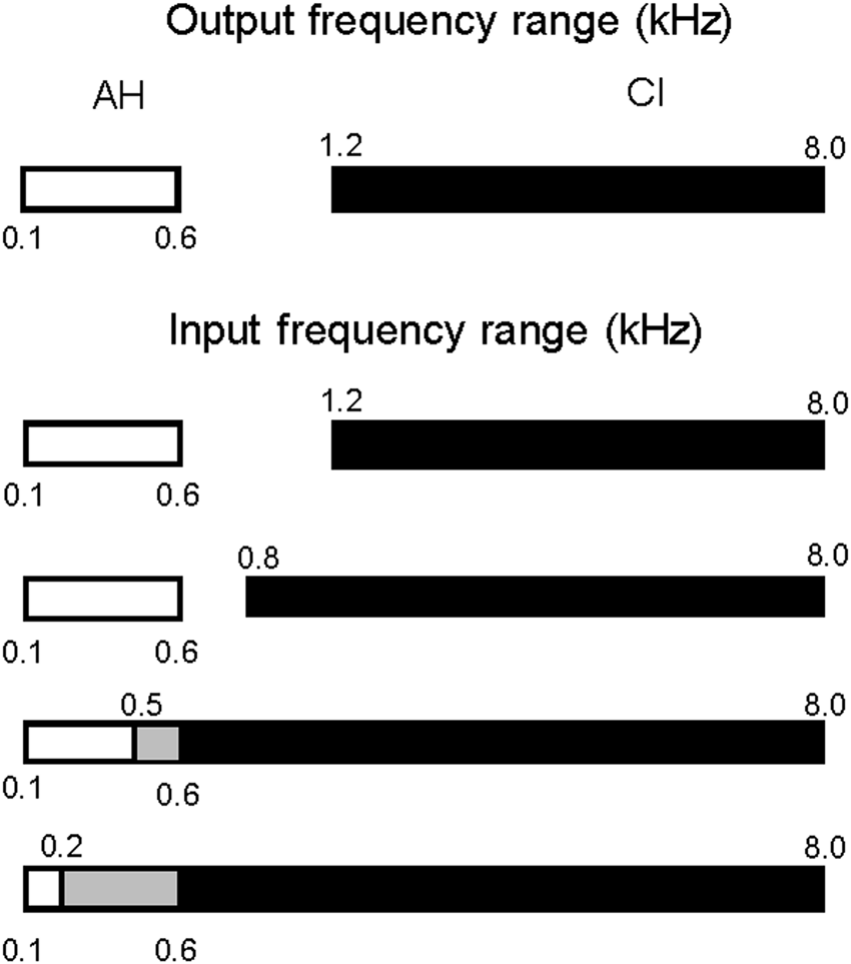
Illustration of the output and input frequency ranges for simulated residual acoustic hearing (AH; white bars) and electric hearing (CI; black bars). The grey bars represent the regions where the AH and CI input frequency ranges overlap.

Figure 1 illustrates the input and output frequency ranges for the simulated residual acoustic hearing (AH) and the 8-channel, noise-vocoded CI simulations. The input and output frequency range for AH was 0.1–0.6 kHz (20^th^ order Butterworth filter; 240 dB/octave). This range was selected to represent residual hearing available to some EAS and bimodal CI listeners, and to convey F1 information for most English vowels. The output frequency range of the CI simulations was 1.2–8.0 kHz. The lowest output frequency (1.2 kHz) corresponds to a cochlear location of a 20-mm insertion of an electrode array according to Greenwood^58^ and is slightly higher than the median upper edge of residual acoustic hearing (approximately 1.1 kHz) for hybrid CI patients reported by Karsten *et al*.^59^. The highest output frequency (8.0 kHz) is similar to the highest input frequency commonly used in commercial CI speech processors. Note that the output range of the CI simulations was not intended to necessarily simulate commercial CI devices, which vary in terms of array length, the number of electrodes, electrode spacing, etc. Rather, the output frequency range was fixed, and the input frequency range was varied to preserve different amounts of acoustic information while introducing different amounts of tonotopic mismatch. The CI input high-cutoff frequency was always 8.0 kHz. The CI input low-cutoff frequency was 0.2, 0.5, 0.8, or 1.2 kHz. When the CI input low-cutoff frequency = 0.2 kHz, there was maximal information within the CI, but with 9.8 mm of frequency mismatch at the apical end of the simulated electrode array; the CI input frequency range also greatly overlapped the AH input frequency range (gray region in Fig. 1), meaning that information between 0.2 and 0.6 kHz would be delivered to different places in the cochlea. When the CI input low-cutoff frequency was 1.2 kHz, there was reduced information within the CI, but no tonotopic mismatch and no overlap between the AH and CI input frequency ranges.

**Figure 2:**
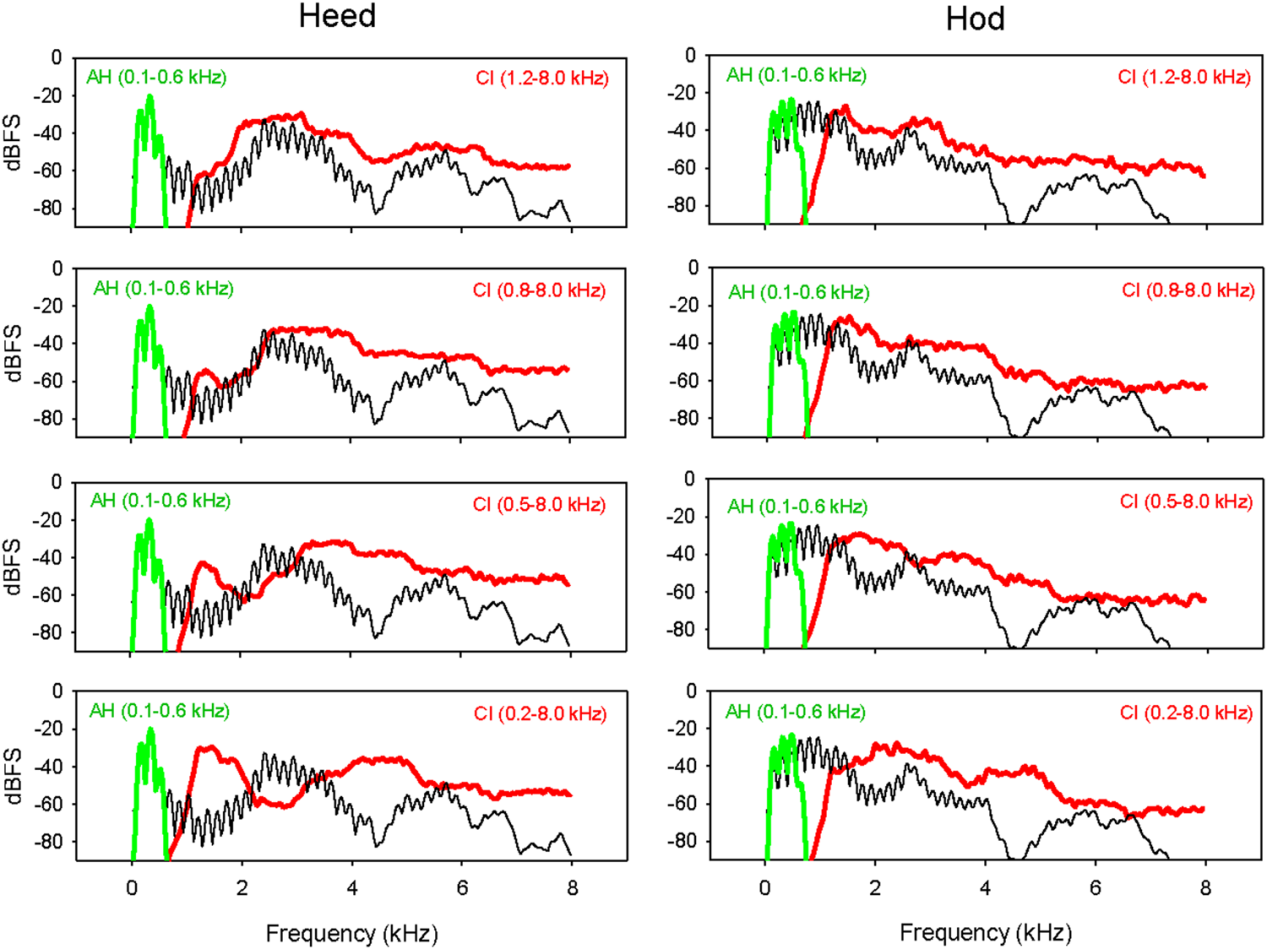
Spectral envelopes for the steady portion of the vowels “heed” (left column) and “hod” (right column). The black lines show the original spectral envelope. The green lines show the spectral envelope with the simulated residual acoustic hearing (AH); the input and output frequency range was 0.1–0.6 kHz. The red lines show the spectral envelope with the CI simulations; the output frequency range was 1.2–8.0 kHz and the input frequency range was varied to preserve different amounts of speech information while introducing different amounts of tonotopic mismatch.

Figure 2 shows spectral envelopes for the vowels “heed” and “hod” produced by male talker 1. For AH (green lines), the original spectral envelope (black lines) is well-preserved within the limited input/output frequency range. When the CI input low-cutoff frequency = 1.2 kHz (red lines in top panels), the tonotopic place is correct and the original spectral envelope is coarsely preserved. However, speech information between 0.6 and 1.2 kHz is completely lost. As the CI input low-cutoff frequency is reduced, more speech information is preserved but is shifted toward the base of the cochlea.

**Figure 3:**
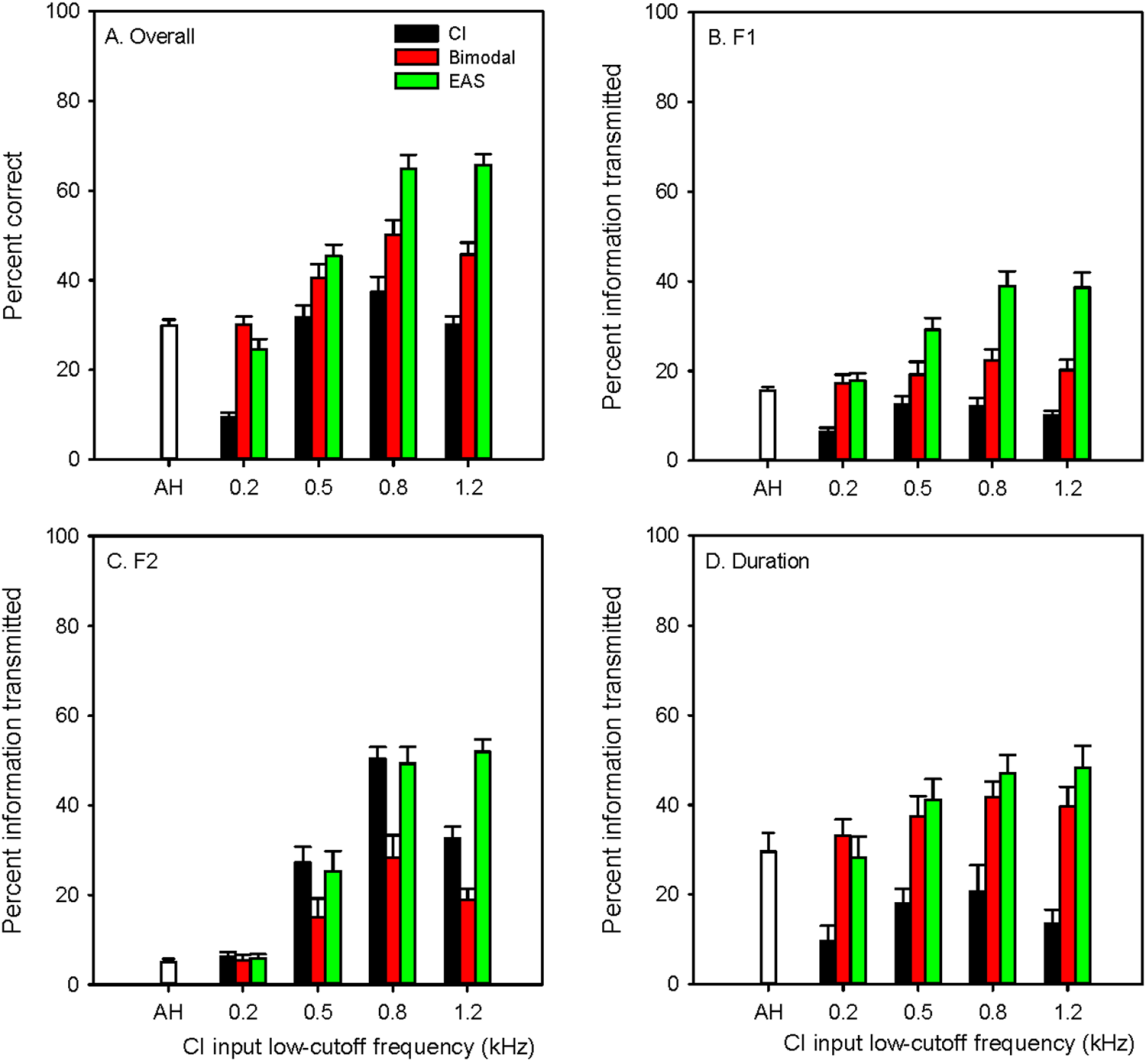
Mean percent correct (N = 10) for overall vowel recognition (**A**), F1 (**B**), F2 (**C**), and duration (**D**). The white bars show performance with simulated residual acoustic hearing (AH), the black bars show performance with the CI simulations alone, the red bars show performance with bimodal listening, and the green bars show performance with EAS. Performance for the CI simulations alone, bimodal, and EAS are shown as a function of the CI input low-cutoff frequency. The error bars show the standard error of the mean.

Vowel recognition was measured for the AH and CI simulations alone (unimodal listening in a single ear), as well for simulated EAS (AH + CI in the same ear) and bimodal listening (AH + CI in opposite ears). Performance was analyzed in terms of overall percent correct, as well as production-based categories of F1, F2, and duration^60^. With the original, unprocessed vowel stimuli, mean scores (across subjects) were 90.4 (SE = 0.8), 92.4 (SE = 2.1), 78.3 (SE = 1.9) and 82.1 (SE = 3.5) percent correct for overall vowel recognition, F1, F2, and duration, respectively. Figure 3 shows mean performance (across subjects) with AH alone, the CI simulations alone, bimodal, and EAS in terms of percent correct for overall vowel recognition, F1, F2, and duration. In general, performance was best for EAS and poorest when the CI input low-cutoff frequency was 0.2 kHz. Two-way repeated measures analyses of variance (RM ANOVAs) were performed on the data shown in each panel in Fig. 3, with simulation (AH, CI simulations with 0.2, 0.5, 0.8, and 1.2 input low-cutoff frequency) and listening condition (unimodal, bimodal, EAS) as factors; results are shown in Table 1.

**Table 1:** Results of two-way RM ANOVAs performed on the data in each panel in Fig. 3.

	Simulation			Mode			Simulation x Mode			post-hoc (p < 0.05)
	dF, res	F	p	dF, res	F	p	dF, res	F	p	
Overall	4, 72	54.7	<*0.001*	2, 72	76.2	<*0.001*	8, 72	30.8	<*0.001*	0.2–1.2: Bi, EAS > Uni; 0.5.−1.2: EAS > Bi Uni: AH, 0.5–1.2 > 0.2 Bi: 0.5–1.2 > AH, 0.2; 0.8 > 0.5 EAS: 0.5–1.2 > AH, 0.2; 0.8–1.2 > 0.5
F1	4, 72	17.3	<*0.001*	2, 72	45.6	<*0.001*	8, 72	18.2	<*0.001*	0.5–1.2: Bi, EAS > Uni; 0.5–1.2: EAS > Bi Uni: AH > 0.2 Bi: 0.8 > AH EAS: 0.5–1.2 > AH, 0.2; 0.8–1.2 > 0.5
F2	4, 72	78.6	<*0.001*	2, 72	21.1	<*0.001*	8, 72	15.1	<*0.001*	0.5–1.2: Uni, EAS > Bi; 1.2: EAS > Uni Uni: 0.5–1.2 > AH, 0.2; 0.8 > 0.5 Bi: 0.8–1.2 > AH, 0.2; 0.8 > 0.5 EAS: 0.8–1.2 > AH, 0.2; 0.8 > 0.5
Duration	4, 72	5.8	*0.001*	2, 72	98.2	<*0.001*	8, 72	9.1	<*0.001*	0.2–1.2: Bi, EAS > Uni; 1.2: EAS > Bi Uni: AH > 0.2–0.5, 1.2 Bi: 0.8 > AH EAS: 0.8–1.2 > AH, 0.2

The far right column shows significant differences from post-hoc Bonferroni pairwise comparisons. Italics show significant *p* values (<0.05). F1 = first formant; F2 = second formant; dF = degrees of freedom; res = residual error; 0.2, 0.5, 0.8, and 1.2 = low input frequency for the CI simulations; AH = residual acoustic hearing simulation; Uni = simulated unimodal listening; Bi = simulated bimodal listening; EAS = simulated electric-acoustic stimulation in the same ear.

The pattern of results was similar for overall vowel recognition and F1 perception. For unimodal listening, performance was significantly poorer only when the CI input low-cutoff frequency was 0.2 kHz; performance was similar but generally poor among the remaining simulations. Bimodal and EAS performance was significantly better than with AH or with the CI simulations when the input low-cutoff frequencies ≥0.5 kHz. Bimodal and EAS performance significantly worsened for CI input low-cutoff frequencies ≤0.5 kHz.

For perception of F2 cues, CI-only performance was best when the input low-cutoff frequency was 0.8 kHz; performance was very poor with AH (due to information loss above 0.6 kHz) and with the CI simulation when the input low-cutoff frequency was 0.2 kHz. EAS performance was significantly better than CI-only when the CI input low-cutoff frequency was 1.2 kHz; there was no significant difference in performance between EAS and CI-only when the CI input low-cutoff frequency was 0.5 or 0.8 kHz. There was no significant difference in EAS performance between the 0.8 and 1.2 kHz CI input low-cutoff frequencies. Strikingly, bimodal performance was significantly poorer than CI-only for CI input low-cutoff frequencies ≥0.5 kHz.

For perception of duration cues, unimodal performance was significantly better with AH than with the CI simulations when the input low-cutoff frequency was 0.2, 0.5, or 1.2 kHz. Bimodal and EAS performance was significantly better than CI-only performance at all input low-cutoff frequencies. EAS performance was significantly better than AH for CI input low-cutoff frequencies ≥0.5 kHz; bimodal performance was significantly better than AH only when the CI input low-cutoff frequencies was 0.8 kHz. EAS performance was significantly better than bimodal only when the CI input low-cutoff frequencies was 1.2 kHz.

**Figure 4:**
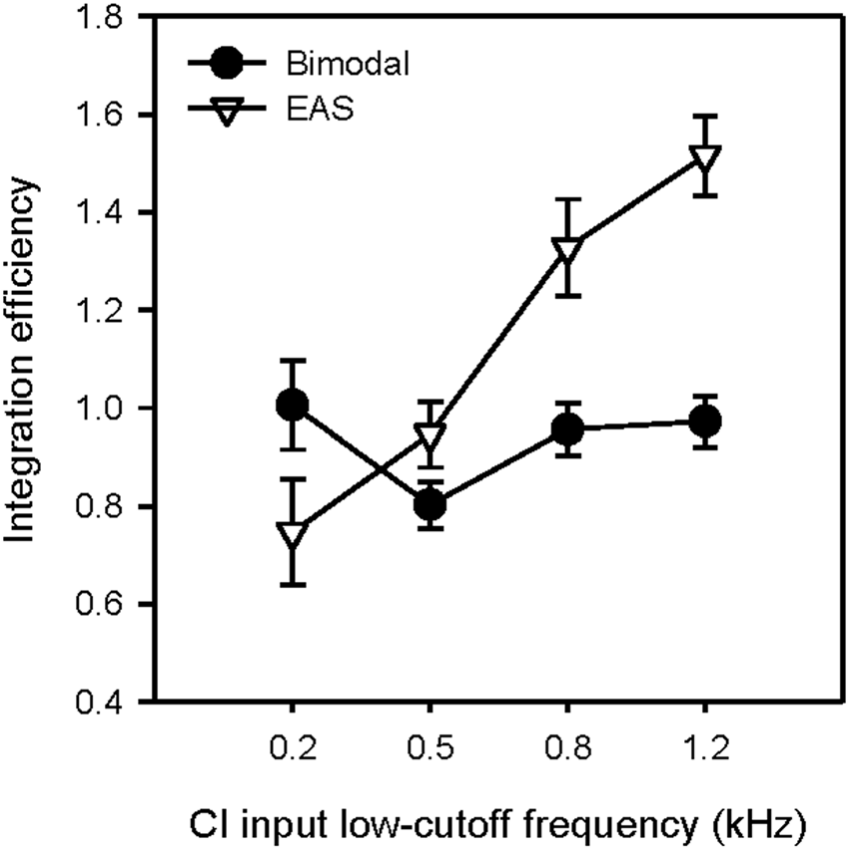
Mean integration efficiency (N = 10) for bimodal (filled circles) and EAS simulations (open triangles) as a function of the CI input low-cutoff frequency. The error bars show the standard error of the mean.

Integration efficiency (IE) for acoustic-electric hearing was calculated for the present bimodal and EAS data as the ratio between the observed and the predicted acoustic-electric hearing performance $({P}_{AH}+{P}_{CI}-{P}_{AH}\,\ast \,{P}_{CI})\,$ ^27^. Figure 4 shows IE for simulated bimodal and EAS, as a function of the CI input low-cutoff frequency; values > 1 imply “super-additive” or synergistic integration of acoustic and electric hearing. A two-way RM ANOVA was performed on the data shown in Fig. 4, with listening condition (bimodal, EAS) and CI input low-cutoff frequency (0.2, 0.5., 0.8, 1.2 kHz) as factors. Results showed significant effects for listening condition (F(1, 27) = 10.0, p = 0.001) and CI input low-cutoff frequency (F(3, 27) = 12.9, p < 0.001); there was a significant interaction (F(3, 27) = 30.4, p < 0.001). Post-hoc Bonferroni pairwise comparison showed significantly better IE with the 0.8 and 1.2 kHz CI input low-cutoff frequencies than with 0.2 or 0.5 kHz (p < 0.05 in all cases). For bimodal IE, there was no significant effect of CI input low-cutoff frequency. For CI input low-cutoff frequencies ≥0.8 kHz, IE was significantly better with EAS than with bimodal (p < 0.05 in both cases). When the CI input low-cutoff frequency was 0.2 kHz, IE was better with bimodal than with EAS.

## Discussion

Contrary to our hypothesis, vowel recognition was much better with simulated EAS than with simulated bimodal listening. With no tonotopic mismatch (CI input low-cutoff frequency = 1.2 kHz), acoustic and electric hearing was more effectively combined in the same ear than across ears. The benefit with EAS (relative to CI-only performance) was 35.1, 26.7, 19.3, and 34.9 percentage points for overall vowel recognition, F1, F2, and duration cues, respectively. The benefit with bimodal (relative to CI-only performance) listening was considerably less: 15.6, 10.1, −13.8, and 26.1 percentage points for overall vowel recognition, F1, F2, and duration cues, respectively. Even with slight tonotopic mismatch (CI input low-cutoff frequency = 0.8 kHz), benefits were much larger for EAS than for bimodal listening.

While acoustic and electric hearing was effectively combined with either EAS or bimodal listening for CI input low-cutoff frequencies ≥0.5 kHz, IE was significantly better with EAS than with bimodal hearing. Previous probabilistic models of combined acoustic-electric hearing assumed that AH and CI information were independent^27,53^; in those studies, the models made no distinction between EAS and bimodal listening. Differences in mechanisms - combining *information* across ears versus combining *energy* within the same ear - may explain differences in IE. With bimodal listening, subjects had to combine independent information from separate ears and separate frequency regions with differing spectral resolution. With EAS, AH and CI information was not truly independent, but rather fused into a stimulation pattern that included detailed spectral envelope information ≤0.6 kHz and coarse envelope information ≥1.2 kHz.

Somewhat consistent with our hypothesis, the degree of tonotopic mismatch significantly affected IE for EAS, but not for bimodal listening. The effect of tonotopic mismatch within electric hearing was not captured in previous studies examining integration of acoustic and electric hearing^27,53^. With the CI alone or bimodal listening, there was very little difference in vowel recognition for CI input low-cutoff frequencies between 0.5 and 1.2 kHz, which explicitly shows the tradeoff between the amount of speech information and the degree of tonotopic mismatch. With EAS, no such tradeoff was observed, as performance was best with either the 0.8 or 1.2 kHz CI input low-cutoff frequencies, and dropped sharply for CI input low-cutoff frequencies ≤0.5 kHz. This suggests that bimodal listeners are able to more efficiently integrate increased amounts of speech information (and the associated increased tonotopic mismatch) from electric hearing with residual acoustic hearing. Conversely, EAS listeners are able to more efficiently integrate acoustic and electric stimulation patterns with small amounts of tonotopic mismatch. EAS performance was much better when the CI input low cut-off frequency was ≥0.8 kHz, despite the information loss. This finding is different from that of Karsten *et al*.^59^, who reported better consonant recognition in real EAS patients when the CI input low cut-off frequency was adjacent to the extent of acoustic hearing. Vowel recognition has been shown to be more sensitive to tonotopic mismatch than consonant or sentence recognition^30^. Again, depending on the position of the intra-cochlear electrodes and the frequency allocation, there may be tonotopic mismatch between the acoustic input and place of stimulation. The present EAS simulation data suggest that tonotopic mismatch had a more deleterious effect on vowel recognition than did the information loss between 0.6 and 1.2 kHz. It is possible that long-term adaptation and/or training might help to accommodate larger mismatches, in which case EAS listeners can make better use of the additional speech information within electric hearing^35–45^.

Auditory training may only offset some of the deficits associated with tonotopic mismatch^44^. No training was provided in this study; as such, the data represent somewhat acute measures. When the low CI input frequency was 0.2 kHz, mean vowel recognition was 9.4, 30.1, and 24.6% correct for CI-only, bimodal and EAS, respectively. Previous training studies show a 10–15 percentage point improvement with training^37,39^. With a 15-point improvement with training, mean scores might have improved to 24.4, 45.1, and 39.6% correct for CI-only, bimodal and EAS, respectively. For EAS, such performance would have remained well below the 65.7% correct observed when the low CI input frequency was 1.2 kHz (tonotopically matched). Interestingly, Zhang *et al*. observed that mean speech performance in EAS listeners improved by approximately 10 percentage points after training with EAS listening for 20 hours^61^. The present data suggest that for EAS patients, the best vowel recognition may be observed when the tonotopic mismatch in the CI is minimized, even when training benefits are considered.

As indications for cochlear implantation continue to expand, and as surgical techniques and electrode designs continue to improve, combining acoustic and electric hearing in the same ear, opposite ears, and even both ears will become more commonplace. Depending on the stability of residual acoustic hearing and/or the insertion depth, complete or partial acoustic hearing preservation may be possible in the implanted ear^62–66^. The present results suggest that minimizing tonotopic mismatch within electric hearing may increase the benefit of acoustic-electric hearing, especially for EAS. Radiological imaging may help to identify the position of the electrodes within the cochlea and identify the extent of acoustic hearing^67,68^. In the clinic, the input low-cutoff frequency for the CI is generally set to be adjacent to the high edge of residual acoustic hearing, as frequency gaps between acoustic and electric hearing have been shown to be detrimental^69^. The present data suggest that tonotopic mismatch may greatly affect EAS performance as well acoustic-electric integration efficiency. Depending on the position of the intra-cochlear electrodes, adjusting the CI input low-cutoff frequency to be adjacent to the extent of acoustic hearing may introduce detrimental tonotopic mismatch. Again, experience with the CI and/or training may help to accommodate this mismatch^35–47^, but minimizing tonotopic mismatch may be preferable for initial CI fitting, possibly followed by progressive widening of the CI input frequency range^45^. Clinical fitting of bimodal CI patients is generally performed without regard to the extent of contralateral acoustic hearing; as such, speech information is maximized within the CI, often with some degree of tonotopic mismatch. The present bimodal results showed a tradeoff between speech information and tonotopic mismatch, and bimodal integration efficiency was not affected by tonotopic mismatch. Still, the best bimodal performance was observed with an optimal tradeoff between speech information and tonotopic mismatch (CI input low-cutoff frequency = 0.8 kHz).

## Conclusions

The present study examined vowel recognition in NH subjects listening to simulations of residual acoustic hearing, CI signal processing, bimodal, and EAS. Key findings include:

1. Simulations of residual acoustic hearing and electric hearing were better combined in the same ear (EAS) than across ears (bimodal). Relative to CI-only, benefits of acoustic-electric hearing were nearly twice as large for EAS than for bimodal listening.
2. Acoustic-electric integration efficiency was generally better for EAS than for bimodal listening. EAS integration efficiency was significantly affected by tonotopic mismatch, while bimodal integration efficiency was not.
3. For CI-only and bimodal listening, there appeared to be an optimal tradeoff between the amount of speech information and tonotopic mismatch. Compared to bimodal listening, EAS was less affected by small amounts of tonotopic mismatch, but more sensitive to larger mismatches.
4. To maximize the benefits of acoustic-electric hearing, CI fitting should minimize tonotopic mismatch, especially for EAS.

## Materials and Methods

This study was approved by the Institutional Review Board of University of California, Los Angeles (UCLA). Prior to participation, written informed consent was obtained from all participants, in accordance with a protocol approved by the Institutional Review Board at University of California, Los Angeles.

### Subjects

Ten NH subjects (4 males and 6 females) participated in this study. The mean age at testing was 40.1 years (range: 18–62 years). All subjects had thresholds <20 dB HL for audiometric frequencies 250, 500, 1000, 2000, 4000, and 8000 Hz.

### Test stimuli and procedure

Multi-talker vowel stimuli were digitized recordings drawn from Hillenbrand *et al*.^70^. Two male and 2 female talkers each produced 16 vowels in a/h-V-d/context (“had,” “hod,” “hawed,” “head,” “heard,” “hid,” “heed,” “hood,” “hud,” “who’,” “hayed,” “hoed”), for a total of 48 stimuli in the set. Stimuli were delivered via circumaural headphones (Sennheiser HDA-200) connected to separate channels of a mixer (Mackie 402 VLZ3), which was connected to an audio interface (Edirol UA-EX). Before signal processing, all stimuli were normalized to have the same long-term root-mean-square (RMS) energy (65 dB).

During testing, a stimulus was randomly selected from the set (without replacement) and presented to the subject, who responded by clicking on one of the 12 response boxed labelled in a/h-V-d/context. No trial-by-trial feedback was provided. Performance was first measured with unprocessed stimuli to familiarize subjects with the task and to ensure high levels of performance before testing with the EAS and bimodal simulations. The remaining test conditions were randomized within and across subjects. Scores were averaged across 2–4 test runs.

### Simulations

Residual acoustic hearing was simulated by bandpass filtering the speech signal between 0.1 and 0.6 kHz (20^th^ order Butterworth filters; 240 dB/octave). CI simulations were 8-channel noise vocoders, similar to Shannon *et al*.^71^. The input frequency range was divided into 8 channels (4^th^ order Butterworth filters; 48 dB/octave), distributed according to Greenwood’s^58^ frequency-to-place formula. The temporal envelope was extracted from each analysis band by half-wave rectification and low-pass filtering (4^th^ order Butterworth filter with 160 Hz envelope cutoff). The temporal envelope from each channel was used to modulate corresponding noise bands; the filter slope for the noise band carriers was same as that of the analysis filters. The modulated noise-bands were summed and the output was adjusted to have the same long-term root-mean-square (RMS) energy as the input speech signal.

Performance with the AH and CI simulations alone (unimodal) were measured with one ear only. For the EAS simulation, AH and CI simulations were delivered to one ear of the headphones. For the bimodal simulation, AH and CI simulations were delivered to opposite ears of the headphones. Unprocessed signals, AH and CI simulations were presented at 60 dBA.

### Availability of materials and data

The data used for the current study are provided as supplementary material.

## Electronic supplementary material

Supplementary Information
